# A systematic review of the effectiveness of antimicrobial rinse-free hand sanitizers for prevention of illness-related absenteeism in elementary school children

**DOI:** 10.1186/1471-2458-4-50

**Published:** 2004-11-01

**Authors:** Emily Meadows, Nicole Le Saux

**Affiliations:** 1 Department of Epidemiology and Community Medicine, University of Ottawa, Ottawa, Ontario, Canada; 2 Division of Infectious Disease, Department of Pediatrics, Children's Hospital of Eastern Ontario, Ottawa, Ontario, Canada

## Abstract

**Background:**

Absenteeism due to communicable illness is a major problem encountered by North American elementary school children. Although handwashing is a proven infection control measure, barriers exist in the school environment, which hinder compliance to this routine. Currently, alternative hand hygiene techniques are being considered, and one such technique is the use of antimicrobial rinse-free hand sanitizers.

**Methods:**

A systematic review was conducted to examine the effectiveness of antimicrobial rinse-free hand sanitizer interventions in the elementary school setting. MEDLINE, EMBASE, Biological Abstract, CINAHL, HealthSTAR and Cochrane Controlled Trials Register were searched for both randomized and non-randomized controlled trials. Absenteeism due to communicable illness was the primary outcome variable.

**Results:**

Six eligible studies, two of which were randomized, were identified (5 published studies, 1 published abstract). The quality of reporting was low. Due to a large amount of heterogeneity and low quality of reporting, no pooled estimates were calculated. There was a significant difference reported in favor of the intervention in all 5 published studies.

**Conclusions:**

The available evidence for the effectiveness of antimicrobial rinse-free hand sanitizer in the school environment is of low quality. The results suggest that the strength of the benefit should be interpreted with caution. Given the potential to reduce student absenteeism, teacher absenteeism, school operating costs, healthcare costs and parental absenteeism, a well-designed and analyzed trial is needed to optimize this hand hygiene technique.

## Background

With the recent emergence of severe acute respiratory syndrome (SARS), a newly discovered infectious disease, the importance of primary infection control measures have been highlighted [1,2]. Routine handwashing with soap and water has been cited by the World Health Organization (WHO) as being "**the most important hygiene measure in preventing the spread of infection**" [3]. This statement has been reiterated by both the United States Centers for Disease Control (CDC) and Health Canada, in reference to reducing the transmission of SARS, the influenza virus, and other infectious pathogens [3-5]. The epidemiological evidence supporting the effectiveness of this basic measure in healthcare settings dates back to the mid-nineteenth century [6,7]. Ignaz Semmelweis, a Hungarian obstetrician, implemented routine handwashing with chlorinated lime by maternity ward staff, as a mechanism to reduce the incidence of puerperal fever [6,7]. This simple routine elicited dramatic results, reducing the mortality rate from 13–18 percent to 2 percent [6,7]. These findings have been replicated numerous times in hospital environments- underlining the magnitude of routine handwashing [8,9].

The elementary school environment is also negatively impacted by outbreaks of disease causing microorganisms [10,11]. These occasional outbreaks result in increased student and teacher absenteeism, increased healthcare expenditures, and an overall decline in the children's learning environment [11]. The United States CDC has estimated that the average school-aged child missed approximately one week annually due to illness-related absenteeism in 1995 [12].

Despite the scientifically proven evidence of the effectiveness of handwashing, and the increasing promotion of proper hand hygiene techniques, observational studies in school settings have indicated that handwashing practices are often lacking [13,14]. Guinan *et al*. (1997) reported that proper handwashing compliance, with soap and water, in school-aged children ranged from 8 to 28 percent. Reported reasons for the observed inadequacy in compliance included insufficient time during the day, and the use of substandard washing facilities in hard to access locations of the school environment [13,14].

In attempts to overcome the obstacles of routine handwashing in school environments, antimicrobial rinse-free hand sanitizers are being used as an alternative hand hygiene technique. The concern is that such programs may be carried out in the absence of evidence of effectiveness in the school environment. Thus, it is timely to review the evidence currently available for the effectiveness of antimicrobial rinse-free hand sanitizer programs in reducing absenteeism due to communicable illness. The aim of this systematic review was to determine whether antimicrobial rinse-free hand sanitizer interventions are effective in preventing illness-related absenteeism in elementary school children.

## Methods

A detailed written protocol was prepared and reviewed in advance (complete protocol can be obtained from the corresponding author).

### Search strategy

A comprehensive search was conducted to identify all relevant studies regardless of publication status. Six electronic databases were searched for studies published in any language. The databases included: Biological Abstracts (1990-May 2003), CINAHL (1982–2003), the Cochrane Controlled Trials Register (1981–2003), EMBASE (1980-May 2003), HealthSTAR (1975-May 2003), and MEDLINE (1966-May 2003). A detailed search strategy was developed for use in MEDLINE and an iterative process was completed to refine the MEDLINE search for each database. Descriptions of the database search strategies are presented in Appendix 1 (see Additional File 1). OVID served as the primary search interface, and the SDI feature was used to monitor newly posted citations, the most recent date September 30, 2004. Due to the low occurrence of studies in this subject area, no filters were used to identify specific study types or reviews.

The reference lists of all relevant articles were reviewed for additional studies. A letter was sent to all corresponding authors of the articles identified by hand-search, excluding two newly eligible citations posted between May 2003 and September 2004 [15,16], or by searching bibliographic databases. Additionally, contact experts and the industrial companies that manufactured the antimicrobial hand gels used in the included trials (GOJO Industries and Woodward Laboratories, Inc.) were contacted in attempts to identify other eligible trials. A detailed list of contacts is provided in Appendix 2 (see Additional File 2). Finally, conference proceedings for the American Journal of Infection Control (2000–2004) and recently published issues of the American Journal of Infection Control (February 2003 to August 2004) were searched by hand.

### Eligibility criteria

Studies were evaluated for inclusion on the basis of four criteria: target population, intervention, outcome, and study design. The target population of interest consisted of elementary school children between 4 and 12 years of age (including senior kindergarten and grades 1 through 8). The interventions of interest were those that administered antimicrobial rinse-free hand hygiene programs compared with no intervention or placebo treatment arm in a school setting. The outcome of interest was the comparison of the number of absences due to communicable illnesses in children who received the antimicrobial rinse-free hand hygiene intervention with the number of such absences in those who received a placebo or no intervention. We included cluster randomized controlled trials (RCTs) and cluster non-randomized controlled trials regardless of publication status.

### Study selection

All relevant citations, titles and abstracts, were imported into a reference database where duplicates were manually removed. Priority in downloading was given to MEDLINE. Reviews were excluded, but the bibliographies from such articles were examined for relevant studies. The screening was completed in an unblinded manner; there is inconclusive evidence that blinding introduced bias into the process [17]. One individual (EM) independently screened the titles and abstracts of each citation and identified all citations for full review. One reviewer was deemed appropriate as it was thought that the reviewer would error on the side of caution. Hard copies of all potentially relevant citations were retrieved, and two reviewers (EM, NLS) independently assessed each article using the aforementioned eligibility criteria, excluding the newly published citations in which eligibility was assessed by EM [15,16]. Disagreements were discussed and a final decision was made by means of open consensus. A pilot test assessing the eligibility criteria on a sample of articles was not performed. Recent studies by both Juni *et al*. 2002 and Moher *et al*. 2003 indicate that the exclusion of trials in languages other than English (LOE) does not bias measures of effectiveness- however, both are cautionary, advocating language inclusive search strategies [18,19]. Due to limited fiscal resources, an English language restriction was applied at this level, however the number of citations in LOE that met eligibility criteria will be noted.

### Data abstraction

Two reviewers (EM, NLS) independently abstracted data from all studies meeting the eligibility criteria, excluding one abstract where EM independently abstracted pertinent information [15], using pre-printed data collection forms presented in Appendix 3 (see Additional File 3). Information pertaining to the descriptive details of the study (e.g., year published, language of publication, publication status), design (e.g., randomized controlled trial), population (e.g., age, grade level), intervention (e.g., type of antimicrobial rinse-free hand sanitizer, inclusion of an educational component), and primary outcome(s) (e.g., absences due to illness) were collected. Adverse advents were not considered due to the relatively benign nature of the intervention. Reviewers resolved differences by means of open consensus. For the case of crossover study designs, data from the both arms of the study was abstracted. A pilot test assessing the data collection form on a sample of articles was not performed.

### Quality assessment

Two reviewers (EM, NLS) independently assessed the quality of each of the included studies, excluding the abstract by Thompson (2004) previously mentioned [15], using the previously validated 3-item Jadad scale, which assesses the quality of the report in terms of randomization generation, double blinding, and withdrawals and drop-outs by intervention group [20]. Studies were not given a quantitative score; rather this was used as a qualitative tool. (Due to the nature of the intervention, not all items apply). Additionally, if trials were randomized, allocation concealment was assessed and qualitatively evaluated as adequate, inadequate or unclear [21]. Disagreements were resolved through open consensus. A pilot study applying the quality assessment criteria on a subset of studies was not completed.

### Data analysis

Data synthesis and analysis was performed in accordance with the Cochrane Reviewers' Handbook [22]. Firstly, data was qualitatively synthesized to examine the overall pattern of studies with respect to study design, population, intervention, and outcome characteristics. Sources of clinical and statistical heterogeneity were identified and results were examined. All data was abstracted as reported. The primary outcome, frequency of absences due to communicable illness was analyzed. Percent relative differences were presented along with 95 percent confidence intervals as the estimate of intervention effectiveness in the four studies which calculated rate and risk ratios as the measure of association [10,27,31,41]. Percent relative differences and 95 percent confidence intervals were calculated enabling the results to be compared between studies, without altering the measure of association reported in the studies (rate and risk ratios). In the studies where data could not be abstracted, measures of association were reported [15,16]. The validity of performing a quantitative synthesis was considered, however based on a qualitative inspection of heterogeneity and estimates of intervention effectiveness this was not deemed appropriate. Thus, sensitivity and subgroup analyses were not performed, and publication bias was not assessed quantitatively.

## Results

### Description of studies

**Figure 1:**
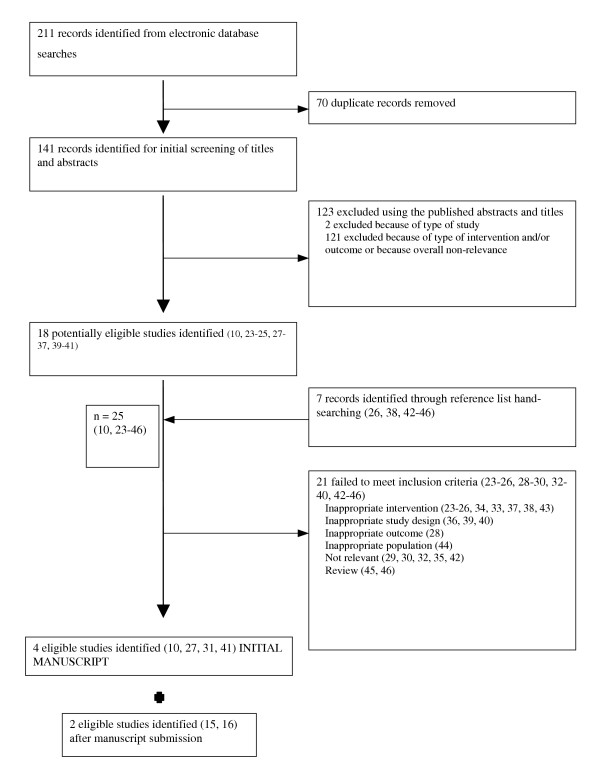
Flow diagram outlining the results of literature search and review of studies retrieved

A flow diagram of the search results is illustrated in Figure 1. From the searches of the electronic databases, a total of 211 citations were identified, of which 70 were duplicates, resulting in the identification of 141 unique citations. In all, 18 potentially relevant trials were retrieved from the searches of the relevant databases. Hand-searching of the reference lists of relevant articles and conference proceedings resulted in a further 7 trials, which were also reviewed for consideration. Thus, 25 studies were determined potentially relevant [10,23-46]. Using both titles and abstracts, no trials meeting the inclusion criteria in LOE were found during the study selection phase.

After review of the full text of these studies, 21 were excluded for the following reasons: no outcomes of interest (n = 1), inappropriate population (n = 1), inappropriate interventions (n = 9), inappropriate study design (n = 3), irrelevant subject matter (n = 5), and review (n = 2). Thus, a total of 4 trials fulfilled the inclusion criteria [10,27,31,41]. However, during the time between manuscript submission and revision, 2 additional citations were deemed to be eligible [15,16], bringing the total to n = 6 eligible studies, one of which was a published manuscript [16], and the other a published abstract [15].

Of the 6 remaining studies, 2 were crossover studies [16,27], 1 was a placebo-controlled cluster randomized controlled trial (RCT) [41], 2 were cluster non-randomized controlled trials (NRCT) [10,31], and the published abstract was a cluster trial, however randomization was unclear [15]. McNemars's test was used to assess observer agreement, chi-square = 2.00; df = 1, p = 0.1573; and there was 92 percent agreement between the two reviewers with respect to study relevance for the initial four trials included [10,27,31,41]. All of the relevant trials are described in Table 1, and an assessment of their quality of reporting is presented in Table 2 (the study by Thompson 2004 was excluded as only the abstract was available). Our overall agreement was 89 percent with respect to data collection for the five included studies outlined in Table 1. For quality abstraction, percent agreement was 80 percent. When examining items relating to blinding and assessment of withdrawals and dropouts, observer agreement was 100 and 80 percent, respectively. Kappa statistics were not calculated as sample size was insufficient.

**Table 1:** Characteristics of studies included in the systematic review, demographics and descriptive statistics

**Author(s), Year, and Country**	**Source of Funding**	**Study Population**	**Definition Illness-related Absenteeism**	**Unit of analysis**	**Study Duration**	**Intervention Arm**	**Control Arm**
**Cluster Randomized Controlled Trials (RCTs)**							
White ^41 ^2001 United States	Industry (Woodward Laboratories, Inc.)	1 private and 2 public elementary schools grades K-6; 72 initial classes (n = 1626 students) * Target group: • 16 classes, n = 388 students Control group: • 16 classes, n = 381 students	• GI or respiratory-related • Parents reported events	Grouped by classroom	5 weeks	• Education: presentation and video describing germs and proper handwashing techniques (1 hr session) • Large involvement of both parents and school staff • Each child received 1-oz bottle of SAB (CleanHands^®^) (alcohol-free) instant hand sanitizer • Instructed to use spray under teacher supervision to supplement handwashing	• Education: presentation and video describing germs and proper handwashing techniques (1 hr session) • Large involvement of both parents and school staff • Each child received 1-oz bottle of placebo formulation • Instructed to use spray under teacher supervision to supplement handwashing
**Crossover**							
Dyer ^27 ^2000 United States	Industry (Woodward Laboratories, Inc.)	Private elementary school (K-6); 2 classrooms per grade level, n = 30 students per classroom Target group: • Children grades K-6 • 1 classroom per grade level • n = 210 students Control group: • Children grades K-6 • 1 classroom per grade level • n = 210 students	• GI (symptoms including vomiting, abdominal pain, and diarrhea) • Respiratory-related (symptoms included cough, sneezing, sinus trouble, bronchitis, fever alone, pink eye, headache, mononucleosis, and acute exacerbation of asthma) • Parents reported events	Grouped by classroom	10 weeks (4 weeks first arm, 2 week washout period, 4 weeks second arm)	• Education: presentation and video describing germs and proper handwashing techniques (1 hr session) • Each child received 1-oz bottle of SAB (CleanHands^®^) (alcohol-free) instant hand sanitizer • Instructed to use spray under teacher supervision to supplement handwashing	• Education: presentation and video describing germs and proper handwashing techniques (1 hr session) • Instructed to wash hands as usual
Morton ^16 ^2004 United States	Maine Administrative School District #35 in Eliot, and South Berwick, Maine; Erie Scientific donated AlcoSCRUB^®^	1 elementary school in northern New England, grades K-3; 17 classrooms and n = 285 students eligible PHASE 1: Target group: • 9 classrooms Control group: • 8 classrooms • PHASE 2: • reversed	• GI (symptoms including influenza, diarrhea, nausea, or vomiting (with or without fever)) • Respiratory-related (symptoms included nasal congestion, cough, or sore throat (with or without fever)) • Parents reported events	Grouped	100 days (46 day first arm, 1 week washout period, 47 day second arm)	• Education: guardians provided with study information and a contact number for the school nurse; additionally, monthly updates were provided • Education: students received a carefully planned education program; 45 minute "Germ Unit", Glo Germ™ presentation • Reinforcement: 1^st ^week: daily reminders given to students, after 1^st ^week reinforcement given weekly and after holidays; each classroom visited twice by school nurse during two arms • AlcoSCRUB^® ^dispensers were furnished in each classroom, located near the classroom entrance at a height that was accessible to all students • Gel use was monitored, and reinforcement was given to classes with low use	• Education: information was presented by school nurse about proper handwashing • Instructed to wash hands as usual
**Cluster Non-randomized Controlled Trials (NRCTs)**							
Hammond ^31 ^2000 United States	GOJO Industries, Inc.	18 public elementary schools grades K-6 in 6 school districts * Target group: • 5 school districts: District 1 (Ohio; K-5; n = 1440 students), District 2 (Ohio; 2,3; n = 266 students), District 3 (Delaware; 3,4; n = 110 students), District 4 (Tennessee; K-6; n = 680 students), District 5 (California; K-5; n = 579 students) Control group: • 5 school districts: District 1 (K-5; n = 1136 students), District 2 (2,3; n = 552 students), District 3 (3,4; n = 113 students), District 4 (K-6; n = 592 students), District 5 (K-5; n = 612 students)	• Infectious process such as cold, flu, and gastroenteritis (common infectious illnesses such as pink eye, abscesses, and skin infections were not included) • No information regarding reporting of events	Grouped by school and grouped by classroom	10 months	• Each test classroom was equipped with a dispenser of PURELL instant alcohol-based hand sanitizer; also placed in other locations around the school • Reinforcement by study co-ordinators every 3 months	• No intervention
Guinan ^10 ^2002 United States	GOJO Industries, Inc.	5 elementary schools; 4 schools had 4 classrooms, 1 school had 2 classrooms (coed and single sex schools) Target group: • Children grades K-3 • 4 schools with 2 classrooms, 1 school with 1 classroom • n = 145 students Control group: • Children grades K-3 • 4 schools with 2 classrooms, 1 school with 1 classroom • n = 145 students	• Infectious process such as cold, flu, and gastroenteritis • Children and parents reported events • Had to be 5 days between episodes to count	Grouped by classroom	3 months	• Education: presentation and video describing germs and proper handwashing techniques (1 hr session) • Each test classroom was equipped with a dispenser of an alcohol-based hand sanitizer	• No intervention
**Cluster Controlled Trials-randomization unclear**							
Thompson ^15 ^2004 United States (abstract only)	Not available	5 grade two classrooms, and 1 one/two combination classroom, n = 138 children Target group: • 3 classrooms Control group: • 3 classrooms	• Illness = cold, flu, conjunctivitis, and gastrointestinal symptoms • Teachers recorded absences	n/a	n/a	• Age appropriate interactive learning session • Alcohol-based hand sanitizer installed in intervention classrooms	n/a

* Not including drop-outs or withdrawals

† n/a = not available

**Table 2:** Quality assessment of trials meeting inclusion criteria

**Author(s), Year, and Country**	**Quality Assessment**	**Allocation Concealment**	**Additional Comments**
**Cluster RCT**			
White ^41 ^2001 United States	Study was described as randomized however did not explain method of randomization; participants and study-coordinators were blinded; description of withdrawals and dropouts provided: of the 72 initial classes (1626 students), 32 classes (16 target and 16 control; 769 students) participated (remainder dropped from analysis)	Unclear	Sample size calculation not defined; statistical methods unclear; parents required to sign detailed informed consent form; study reviewed and approved by the two school boards; soap and handwashing not monitored; clustering not accounted for
**Crossover**			
Dyer ^27 ^2000 United States	Study was not formally randomized; neither participants or study-coordinators were blinded; description of withdrawals and dropouts provided: no exclusions from the population were necessary	Not Relevant	Sample size calculation not defined; statistical methods unclear; no parental consent form; study not approved by a formal university institutional review board (approved by school board of education); limited SES diversity; soap and handwashing not monitored; clustering not accounted for
Morton ^16 ^2004 United States	Study was described as randomized however did not explain method of randomization; neither participants or study-coordinators were blinded; description of withdrawals and dropouts provided: of the 17 initial classes (285 students), 17 classes ((253 students, 120 girls and 133 boys), non-consent = 22 children, adverse-events = 10 children)	Unclear	Sample size calculation not defined; data not in a format which could be easily extracted; study approved by Board of Education, and the Institutional Review Board at the state's largest hospital; a consent form was sent to all parents and guardians; clustering not accounted for
**Cluster NRCTs**			
Hammond ^31 ^2000 United States	Study was not formally randomized; neither participants or study-coordinators were blinded; description withdrawals and dropouts provided: 1 school district did not comply with protocol; 25/3080 students did not participate or complete the protocol (in each case, data was not used for results)	Not Relevant	Sample size calculation not defined; no parental consent form; formal review not mentioned; clustering not accounted for
Guinan ^10 ^2002 United States	Study was not formally randomized; participants and study-coordinators were not blinded; description withdrawals and dropouts not provided	Not Relevant	Sample size calculation not defined; statistical methods unclear; no parental consent form; formal review not mentioned (approved by each school); limited SES diversity (high SES); performed in peak absenteeism season; clustering not accounted for

All six trials were conducted in the United States, 4 with reported industrial sponsorship, and were published between 2000 to 2004. The trials varied in size (range = 138 to 6080 students; range = 1 to 18 schools), and geographic locations (Pennsylvania [10], California [27,37], Ohio/Tennessee/Delaware/California [31], New England [16]). One of the studies assessed only private schools [28]; another study assessed both private and public schools [41], three assessed only public schools [10,16,31], while one's type of school assessed was not available [15]. Additionally, there was considerable variation in the type of school included both within and between trials: Christian private school, public elementary schools, same-sex schools and co-ed schools. The duration of the studies ranged from 5 weeks to 10 months, the longest trial being that of the RCT [41].

The trials also varied with respect to the intervention administered. White *et al*. (2001) and Dyer *et al*. (2000), provided each student with alcohol-free instant hand sanitizer [27,41], whilst Hammond *et al*. (2000), Guinan *et al*. (2002), Morton *et al*. (2004), and Thompson *et al*. (2004) provided each class with alcohol-based instant hand-sanitizer dispensers [10,15,16,31]. Education was concurrently provided for both the control and intervention arms in two studies [16,27,41], education on germs and hygiene was provided only to the intervention arm in one study [10], one study did not provide any education however study reinforcement was provided for teaching staff [31], and one study provided education to the intervention arm but as only the abstract was available for this study it was unclear if the control arm received any education [15].

### Methodological quality

The quality of reporting of the 5 trials that were examined in detail was low. Only one study was described as being randomized and double-blinded, however, it failed to describe a detailed and appropriate method of randomization and allocation concealment was unclear [41]. Four of the five studies, as previously mentioned, discussed withdrawals and dropouts however the description was quite basic and detailed flow-diagrams outlining the passage of participants through the trial were not supplied [16,27,31,41]. White *et al*. (2001) reported a significant number of dropouts (857 of 1626 students did not complete the study) however no explanations were offered [41]. Four studies received industrial sponsorship either by GOJO industries or Woodward Laboratories. In addition, two studies received financial support from another external source [16,41]. Other characteristics of poor quality reporting included: no sample size calculation defined for all five studies, and the statistical methods were vague. No studies took into consideration clustering when analyzing their results. Our overall agreement for all items of quality was greater than 80 percent; again observer agreement statistics were not calculated as the sample size was insufficient.

### Primary outcome

All six studies varied in their definition of communicable illness-related absenteeism, refer to Table 1. Out of the five studies with published manuscripts, four of the studies reported the estimate of intervention effectiveness in terms of risk/rate ratios, subsequently calculating percent relative effect, and one reported and odds ratio for a pair-matched study; results are reported in Table 3, Table 4, and Table 5. The percent relative effect measures the decreased rate of the occurrence of absenteeism when the rate ratio is the measure of association or the decreased risk of absenteeism when the relative risk is the measure of association. Tests of significance were completed in all five of the studies using chi-squared tests or t tests however no confidence intervals were calculated. Two of the studies used rate ratios as the measure of association [10,31], two studies used relative risks as the measure of association [27,41], and the other used the odds ratio [16]. Table 3, 4 and 5 distinguish between the measures of association used. All studies found a statistically significant effect of the antimicrobial rinse-free hand gel interventions. In the study by Hammond *et al*. (2000), the experimental group had a 20% (95% CI = 19–21%) reduction in absences due to communicable illness, the experimental group in the trial completed by Guinan *et al*. (2002) had a 49% (95% CI = 42–56%) reduction, White *et al*. (2001) demonstrated a 33% (95% CI = 17–45%) reduction in the experimental group, Dyer *et al*. (2000) had a 34 % (95% CI = 10–50%) reduction in absences due to communicable illness in the experimental group in the first phase and a 56 % (95% CI = 31–72%) in the second phase, and Morton *et al*. (2004) reported a significant odds ratio for McNemar's test (chi-square = 7.787; p = 0.0053).

**Table 3:** Absences due to communicable illness, person-time incidence rates and percent relative effects of a non-alcoholic rinse-free hand sanitizer

			**Intervention**		**Control**		
			
**Trials**		**No. of students**	**No. of absences (no. of student-days)**	**Absenteeism rate per 100 student-days**	**No. of absences (no. of student-days)**	**Absenteeism rate per 100 student-days**	Percent Relative Effect (95% CI)*
White et al. ^41^		770	153 (9615)	1.59	222 (9459)	2.35	33 (17, 45)
Dyer et al. ^27^	Phase 1	420	70 (4136)	1.69	105 (4120)	2.55	34 (10, 50)
	Phase 2	420	28 (4156)	0.674	63 (4140)	1.52	56 (31, 72)

* Percent relative effect = (1- intervention rate/control rate)*100

95 percent confidence interval = (1–95% UCL of Rate Ratio)*100 to (1–95% LCL of Rate Ratio)*100

**Table 4:** Absences due to communicable illness, cumulative incidence rates and percent relative effects of an alcoholic rinse-free hand sanitizer

		**Intervention**		**Control**		
		
**Trials**	**No. of students**	**No. of absences (No. of students)**	**Absenteeism risk**	**No. of absences (No. of students)**	**Absenteeism risk**	Percent Relative Effect (95% CI)*
Hammond et al. ^31^	6080	7441.5 (3075)	2.42	9066 (3005)	3.02	20 (19, 21)
Guinan et al. ^10^	290	140 (145)	0.97	277 (145)	1.91	49 (42, 56)

* Percent relative effect = (1- intervention risk/control risk) *100

95 percent confidence interval = (1–95% UCL of Risk Ratio)*100 to (1–95% LCL of Risk Ratio)*100

**Table 5:** Measures of association reported for studies in which no data could be extracted

**Trials**	**No. of students**	**Raw data reported**	**Measures of association, and statistical tests reported**
Thompson ^15^	138	days absent per student in intervention group = 2.30 days absent per student in control group = 3.20	• Overall reduction in absenteeism due to illness was 28 percent for children using alcohol hand rub
Morton et al. ^16^	253	**N = 211 absent overall** n = 42 never absent due to illness n = 103 ill regardless of participation in the control or the AlcoSCRUB^® ^group n = 69 ill in control group n = 39 ill in AlcoSCRUB^® ^group	• McNemar's test for dichotomous variables with paired subjects, was used to assess strength of intervention: chi-square = 7.787; p = .0053 • Odds of being ill decreased by 43 percent with use of AlcoSCRUB^®^

## Discussion

Many studies have examined the importance of preventing the transmission of infectious diseases in the school environment, one such studied completed by Cramer *et al*. 1999, indicated this item to be of great concern for the parent's of school-aged children [47]. The most common infections transmitted in school environments are respiratory (influenza, pharyngitis etc) and diarrheal illnesses (i.e., Norwalk virus). Most of the infections occur at a constant low level but occasionally outbreaks do occur resulting in increased absenteeism and involvement of public health authorities. Since hands are the primary mechanism of transmission of these illnesses, proper hand hygiene techniques have been endorsed as the first defence at reducing the risk of transmission [1-5,8,10,28]. In health care settings, the routine use of antimicrobial alcohol based hand gels has been endorsed as an alternative to handwashing when hands are not visibly soiled [48-50]. Effectiveness in the hospital setting has not been easy to document given the relative low incidence of documented infections that can be specifically related to nosocomial transmission relative to the high number of handwashing opportunities in specific environments such as intensive care unit settings.

Can the evidence from these six trials reported here be used to promote this type program in elementary schools at the present time? This systematic review of antimicrobial rinse-free hand sanitizers for prevention of illness-related absenteeism in elementary school children is the first review, of the author's awareness, to assess this issue. Although randomized controlled trials are the study design least likely to provide biased estimates of effect, due to the nature of school-based interventions, the inclusion of both randomized and non-randomized cluster controlled trials was required [51]. Of the six studies that met our inclusion criteria, three were non-randomized cluster controlled trials. Recent evidence indicates that non-randomized designs overestimate the effect of an intervention, thus the magnitude of the results should be interpreted with caution [52].

Four of the six studies used an alcohol-based product, the other two using a benzalkonium chloride based disinfectant. The FDA in the United States has indicated that insufficient data exits to classify the latter compounds as safe and effective to use as antiseptic handwashes. They are also adversely affected in the presence of organic material such as food residues, which may be an issue in schools [53]. Four studies were industry sponsored, and five were flawed due to the lack of sample size calculations. The five studies included were of low quality and methodologically weak. The only blinded randomized study using a placebo incorporated in this review was reported to be randomized and double-blinded, however, a description of the randomization technique was not discussed in the report and allocation concealment was unclear [41]. Additionally, this study suffered from a large proportion of withdrawals and drop-outs, thus the results had to be cautiously interpreted. Current studies have indicated that poor quality studies are associated with exaggerated treatment effects [52]. Although all studies reported statistically significant effects of the antimicrobial rinse-free hand gel in the experimental group, the aforementioned evidence suggests the reader should interpret these results cautiously. Thus, a clear delineation of the effectiveness of the intervention cannot be resolved from this review.

Several limitations were encountered when completing this review, the major one being the scarcity of high quality studies. Additionally, although content experts, primary authors and industrial companies were contacted, no grey literature was found. The possible existence of unpublished non-significant trials should not be discounted. The validity of performing a quantitative synthesis was considered, however based on a qualitative inspection of heterogeneity and estimates of intervention effectiveness this was not deemed appropriate. Sources of heterogeneity included study designs, population characteristics, intervention characteristics, case definition and primary outcome measure. Thus, sensitivity and subgroup analyses were not performed, and publication bias was not assessed quantitatively. Another limitation was the fact that one reviewer was used to do the broad screen of articles and review the two citations identified between September 2003 and the present time. This may have biased the results; however, it is believed that this reviewer would overestimate the citations to be included.

## Conclusions

In wake of the recent worldwide emergence of Severe Acute Respiratory Syndrome (SARS), the importance of proper hand hygiene has been brought to the spotlight. Comprehensive hand hygiene programs with occasional reinforcement are an inexpensive intervention, which potentially can work for a broad population, with minimal adverse effects. Future research should concentrate on developing study protocols that are scientifically sound with regards to randomization generation, blinding, allocation concealment and other factors that will minimize or avoid bias. Hand hygiene programs are the most important infection control measure in the school environment and have potentially large public health and economic implications therefore their design, implementation, and analysis should be carried out with the rigour.

## Competing interests

The author(s) declare that they have no competing interests.

## Authors' contributions

EM conceived and designed the study as part of a graduate course in systematic reviews, reviewed trials for inclusions, abstracted data, participated in data analysis, and drafted and revised the manuscript.

NLS participated in initial study design, reviewed trials for inclusion, abstracted data, participated in data analysis, and revised the manuscript.

Both EM and NLS agreed upon the final revision.

## Appendices

Appendix 1 – Syntax for searches

Appendix 2 – List of corresponding authors, content experts and industrial companies contacted

Appendix 3 – Data collection form

## Pre-publication history

The pre-publication history for this paper can be accessed here:



## Supplementary Material

Additional file 1 Additional file 1 - Syntax for searches Click here for file

Additional file 2 Additional file 2 - List of corresponding authors, content experts and industrial companies contacted Click here for file

Additional file 3 Additional file 3 - Data collection form Click here for file
