# Supplementary material for: A systematic review of the effectiveness of antimicrobial rinse-free hand sanitizers for prevention of illness-related absenteeism in elementary school children
Source: BMC Public Health. 2004 Nov 1;4:50. doi: 10.1186/1471-2458-4-50 (PMC534108; doi:10.1186/1471-2458-4-50)
Supplement: Additional file 1 — Syntax for searches [file 1471-2458-4-50-S1.DOC]

# Appendix 1:

Syntax for literature searches

**Biological Abstracts**

1. absenteeism.sh.
2. attendance.sh.
3. Infection.sh.
4. ((elementary or primary) adj (school or public)).mp. [mp=title (english), book title (english), original language book title (non-english), abstract, subject headings, concept codes, biosystematic codes/super taxa, heading words]
5. nursery school.mp.
6. (day care or daycare or day-care).mp. [mp=title (english), book title (english), original language book title (non-english), abstract, subject headings, concept codes, biosystematic codes/super taxa, heading words]
7. ((handwash$ or hand) adj (wash$ or sanit$ or hygiene)).mp. [mp=title (english), book title (english), original language book title (non-english), abstract, subject headings, concept codes, biosystematic codes/super taxa, heading words]
8. 1 or 2 or 3
9. 5 or 6
   10. 4 not 9
   11. 10 and (7 or 8)
   12. 10 and (1 or 2 or 7)

**Cochrane Controlled Trials Register**

1. (hand and (washing or hygiene or sanitation)).mp. [mp=ti, ab, tx, kw, ct, ot, sh, hw]

2. ((elementary or primary) and (school or public)).mp. [mp=ti, ab, tx, kw, ct, ot, sh, hw]

3. 1 and 2

4. absenteeism.mp. [mp=ti, ab, tx, kw, ct, ot, sh, hw]

5. 2 and 4

**CINAHL**

1. exp ABSENTEEISM/

2. exp Communicable Diseases/ or Infection/

3. exp Schools, Elementary/ or exp Students, Elementary/

4. (elementary adj public).mp. [mp=title, cinahl subject headings, abstract, instrumentation]

5. exp Schools, Nursery/

6. exp Day Care/

7. exp handwashing/ or infection control/

8. (hand adj (sanit$ or hygiene)).mp. [mp=title, cinahl subject headings, abstract, instrumentation]

9. 1 or 2

10. 3 or 4

11. 5 or 6

12. 10 not 11

13. 7 or 8

14. 12 and (9 or 13)

**EMBASE**

1. exp ABSENTEEISM/

2. exp COMMUNICABLE DISEASE/ or INFECTION/

3. exp PRIMARY SCHOOL/

4. (elementary adj public).mp. [mp=title, abstract, subject headings, drug trade name, original title, device manufacturer, drug manufacturer name]

5. exp NURSERY SCHOOL/ or DAY CARE/

6. (daycare or day-care or (day adj care)).mp. [mp=title, abstract, subject headings, drug trade name, original title, device manufacturer, drug manufacturer name]

7. exp school hygiene/ or hand washing/ or infection control.mp. [mp=title, abstract, subject headings, drug trade name, original title, device manufacturer, drug manufacturer name]

8. (hand adj (sanit$ or hygiene)).mp. [mp=title, abstract, subject headings, drug trade name, original title, device manufacturer, drug manufacturer name]

9. 1 or 2

10. 3 or 4

11. 5 or 6

12. 10 not 11

13. 7 or 8

14. 12 and (9 or 13)

**HealthSTAR**

1. exp absenteeism/

2. exp communicable diseases/ or infection/

3. ((elementary or primary) adj (school or public)).mp. [mp=title, abstract, keywords, mesh subject heading]

4. exp nursery school/ or day care/

5. exp infection control/

6. ((handwas$ or hand) adj (wash$ or sanit$ or hygiene)).mp. [mp=title, abstract, keywords, mesh subject heading]

7. 1 or 2

8. 3 not 4

9. 5 or 6

10. 8 and (7 or 9)

**MEDLINE**

1. exp ABSENTEEISM/

2. exp Communicable Diseases/ or Infection/

3. (primary school or (elementary adj (school or public))).mp. [mp=title, abstract, cas registry/ec number word, mesh subject heading]

4. exp Schools, Nursery/ or Day Care/

5. exp Handwashing/ or exp Infection Control/

6. (hand adj (sanit$ or hygiene)).mp. [mp=title, abstract, cas registry/ec number word, mesh subject heading]

7. 1 or 2

8. 5 or 6

9. 3 not 4

10. 9 and (7 or 8)
