# Supplementary material for: A systematic review of the effectiveness of antimicrobial rinse-free hand sanitizers for prevention of illness-related absenteeism in elementary school children
Source: BMC Public Health. 2004 Nov 1;4:50. doi: 10.1186/1471-2458-4-50 (PMC534108; doi:10.1186/1471-2458-4-50)
Supplement: Additional file 2 — List of corresponding authors, content experts and industrial companies contacted [file 1471-2458-4-50-S2.DOC]

# Appendix 2:

List of corresponding authors, content experts and industrial companies contacted

| Contact | Position | Contact Information |
| --- | --- | --- |
| Dr. M.L. Rotter | Professor of Hygiene and Microbiology  Clinical Institute of Hygiene and Medical Microbiology of the University of Vienna | [hygiene@univie.ac.at](mailto:hygiene@univie.ac.at)  Tel.: 43 1 414 90 79401  Fax: 43 1 404 90 9794 |
| Dr. Elizabeth Henderson | Epidemiologist  Infection Control,  Calgary Health Region | [Elizabeth.Henderson@CalgaryHealthRegion.ca](mailto:Elizabeth.Henderson@CalgaryHealthRegion.ca)  Tel.: 403 943 4027  Fax: 403 291 2571 |
| Dr. Elaine Larson | Professor of Epidemiology  Joseph Mailman School of Public Health  Columbia University  Editor, American Journal of Infection Control | [ell23@columbia.edu](mailto:ell23@columbia.edu)  Tel.: 212 305 0723  Fax : 212 305 0722 |
| Dr. Didier Pittet | Professor of Medicine  Department of Internal Medicine  Infection Control Program  University of Geneva Hospitals | didier.pittet@hcuge.ch |
| Dr. David Dyer | Vice President of Research and Product Development  Woodward Laboratories, Inc. | [dldyer@woodwardlabs.com](mailto:dldyer@woodwardlabs.com)  Tel: 562 598 0010  Fax: 562 598 0010 |
| Brian Hammond† | GOJO Industries Inc. | GOJO Industries, Inc.  PO Box 991  Akron, OH  44309 |
| Dr. Maryanne McGuckin | University of Pennsylvania School of Medicine  Philadelphia | University of Pennsylvania School of Medicine  605A Stellar-Chance Bldg  422 Currie Blvd  Philadelphia, PA  19104-6021 |

# † in process
