# Supplementary material for: A systematic review of the effectiveness of antimicrobial rinse-free hand sanitizers for prevention of illness-related absenteeism in elementary school children
Source: BMC Public Health. 2004 Nov 1;4:50. doi: 10.1186/1471-2458-4-50 (PMC534108; doi:10.1186/1471-2458-4-50)
Supplement: Additional file 3 — Data collection form [file 1471-2458-4-50-S3.DOC]

# Appendix 3:

Data Collection Form

| Title: | | | Unique Identifier No.:  Date of Last Revision: 1 April 03 | | |
| --- | --- | --- | --- | --- | --- |
| Study Characteristics: | | |  | | |
| Descriptive Details: | | |  | | |
| Authors:  Journal: | | |  | | |
| Year Published: | | |  | | |
| Country: | | |  | | |
| Publication Status: | Published | |  | | |
|  | Unpublished | | If yes, details _______________________________ | | |
| Sources of Funding: | Industry | |  | | |
|  | Other | |  | | |
| Where study found? | Database | |  | | |
|  | Other | |  | | |
| Study Design: | | |  | | |
| Type of Research Design | | |  | | |
| Number of schools/classrooms (total) | | |  | | |
| Number of students (total) | | |  | | |
| Duration (months)  Dropouts/withdrawals | | |  | | |
| Population: | | | Intervention | Control | Total |
|  |  |  |  |  |  |
| Schools | | |  |  |  |
| Classes  Students | | |  |  |  |
| Ages  Grades | | |  |  |  |
| Type of School/School Board  Coed/Same sex school  Male/Female Ratio | | |  |  |  |
| Characteristics of Study Intervention: | | | Intervention | Control | Total |
| Type | | |  |  |  |
| (explain) | | |  |  |  |
|  | | |  |  |  |
| Education Included | |  | Yes | Yes |  |
|  |  |  | No | No |  |
| (explain) | |  |  |  |  |
| Primary Outcomes: | | | Intervention | Control | Total |
|  |  |  |  |  |  |
| Detail of primary outcome  Incidence of Illness-Related Absenteeism | | |  |  |  |
